# Experimental study on shear characteristics of the silty clay soil-ice interface

**DOI:** 10.1038/s41598-022-23086-z

**Published:** 2022-11-16

**Authors:** Wanjun Huang, Xuesong Mao, Qian Wu, Linlin Chen

**Affiliations:** 1 Chang an University, Highway Academy, Xian, 7100000 People’s Republic of China; 2 Luoyang Urban Construction Survey and Design Institute Co., Ltd., Luoyang, 471000 People’s Republic of China

**Keywords:** Engineering, Civil engineering, Cryospheric science

## Abstract

During the spring thawing, the decrease of soil-ice interface strength by temperature may lead to slope instability. For this reason, some researchers have explored the relationship between temperature and soil-ice interface strength. However, previous studies have not systematically explored the change law of strength at the soil-ice interface from negative temperature to 0 °C. Therefore, direct shear tests were conducted at different shear temperatures and different moisture contents. The effects of temperature and moisture content on strength, cohesion, and internal friction angle are analyzed, while the shear failure mechanism of specimens at different temperatures is discussed according to the location of the shear failure surface. The results show that: Shear properties of soil ice specimens are related to the unfrozen moisture content. The strength of the sample decreases with increasing temperature, and the change in strength is most significant from − 2 to − 0 °C. The strength reduction in this range is from 21.8 to 74.8%, and the higher the moisture content the more obvious this phenomenon is. The shear index tends to decrease with the increase of unfrozen water content, and the greater the increase of unfrozen water, the faster the decrease of both, especially in stage 2. When the temperature is higher than − 5℃, the failure surface is located above the soil-ice interface, and the strength of the specimen is similar to that of the frozen soil. When the temperature is − 10℃, the shear damage surface appears at the soil-ice interface, and the strength of the specimen is determined by the strength of the soil-ice interface.

## Introduction

The soil-ice interface is a special interface formed by the cementation of soil and ice layers, which is widely distributed in the natural permafrost layer. Due to the special connection and structure between the ice and soil layers, the mechanical behaviors at the interface are largely different from those of general frozen soil. In the past 40–50 years, with the increase in temperature in the Qinghai-Tibet region, slope instability in cold areas has occurred frequently. The decay of the strength of the freeze–thaw and soil-ice interfaces may also be an important factor in slope damage. Therefore, it is necessary to study the shear properties of the soil freeze–thaw interface to understand the influence of the strength change of the soil-ice interface on slope stability.

Researchers have recently conducted numerous studies on slope instability due to active layer melting in permafrost regions^1^. Extensive studies have shown that slope failure in permafrost areas is associated with the thawing of the active layer^1,2^, and the decay of strength at the freeze–thaw interface is the key to causing slope instability^3^. Moisture content and temperature are the two most important factors affecting the strength of frozen soil^4–6^. Meanwhile, a large number of studies have confirmed that the change of strength is mainly realized by changing the state of water through temperature^7–9^.

Therefore, a series of indoor tests have been conducted by researchers to study the variation pattern of freeze–thaw interface strength under different conditions. Peng et al.^10,11^ found that the strength of the freeze–thaw interface was sensitive to the changes in temperature and moisture content through indoor tests. Ge Qi et al.^12^ studied the relationship between freeze–thaw interface shear strength and freeze–thaw cycles under low-temperature conditions. Wang et al.^13^ found that the variation of the strength of the freeze–thaw interface at different moisture contents was mainly related to cohesion. In addition, some researchers have studied the effect of soil type and confining pressure on strength^14^.

In addition, Yazdani et al.^15^ conducted a series of tests with temperature-controlled direct shear apparatus to evaluate the effect of the thermal cycle on the strength of soil–the pile interface. Zhao et al.^16^ conducted a series of tests to analyze the effect of the rising frozen temperature, moisture content, and normal stress on the mechanical properties of the frozen soil-pile interface during the thawing process of permafrost. Janipour et al.^17^ investigate the interface shear strength characteristics between soil and concrete under static loads. Noroozi et al.^18^ evaluated the mechanical behavior of the interface between the filter soil material and the asphalt concrete core in the laboratory.

With the continuous research on the mechanism of strength change of frozen soil freeze–thaw interface and soil-structure interface, researchers have also started to pay attention to the effect of temperature on the strength of the soil-ice interface. Du et al.^19^ used uniaxial tests to research the relationship between strength and water content of ice-rich frozen silty sand. Gao et al.^14^ conducted shear tests at positive temperature and shear tests at the soil-ice interface for different types of soils, respectively, and found that the strength of the soil-ice interface is higher than that of the thawed soil, and the type of soil affects the strength of the freeze–thaw interface. Shi et al.^20^ conducted shear tests at the soil-ice interface at negative temperatures and found that temperature affects shear characteristics.

Most of the past studies are about the freeze–thaw interface and soil-structure interface. Researchers have a preliminary understanding of the mechanical mechanism of freeze–thaw interface and soil-structure surface based on a large number of experiments. The research on the soil-ice interface has also started, and the preliminary analysis of the influence of internal and external factors on the shear properties of the soil-ice interface has been carried out. Although some studies have explored the effects of temperature, initial moisture content, etc. on the strength of the soil-ice interface, these studies have not systematically investigated the development of the strength of the soil-ice interface at different temperatures. In addition, the experimental studies on the soil-ice interface are still relatively few, and the influence of the change of soil-ice interface strength on the stability of permafrost slopes and the shear characteristics of the soil-ice interface still needs further research.

Therefore, this paper investigates the change law of soil-ice interface strength during the thawing process by improving the test method and conducting a controlled temperature direct shear test on the soil-ice specimens. The direct shear test of the soil-ice interface was carried out at different initial moisture contents (9.0, 16.0, 23.0%), different temperatures (− 10, − 5, − 2, − 1, 0 °C), and different normal stresses (100, 200, 300, 400 kPa). The variation laws of shear strength and shear index with temperature and moisture content were revealed, and the mechanism of shear strength decay of specimens during the thawing process was discussed. In addition, the changes in the location of the shear failure surface during the thawing process were also analyzed. And these discussions and analyses are of great significance for understanding the mechanical decay mechanism of the soil-ice interface, and also can provide some theoretical basis for the study of slope stability in a cold region.

## Material and method

### Soil sample

**Figure 1:**
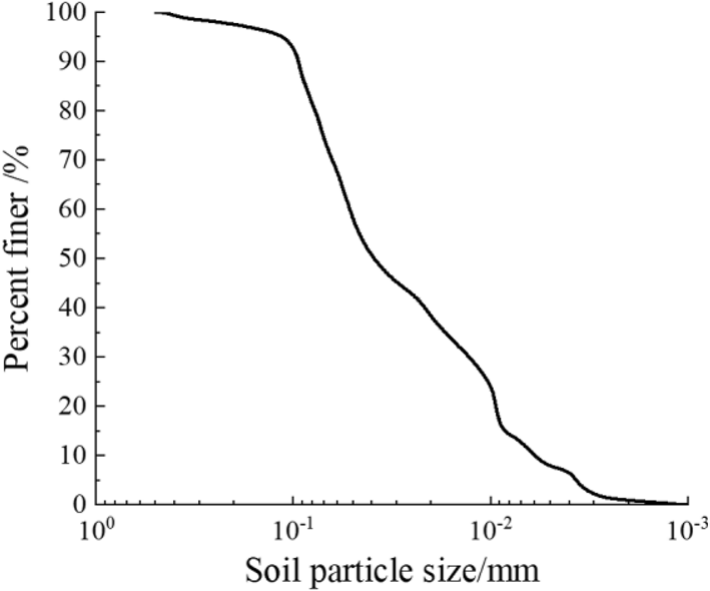
Particle grading curve.

The soil, used in the direct shear test, is silty clay, which was all taken from a slope at the Qinghai-Tibet plateau. The grain size distribution and physical properties of the soil are shown in Fig. 1 and Table 1.

**Table 1:** Basic physical properties of the soil sample.

Soil	Grain density/(g/cm^3^)	Liquid limit/%	Plastic limit/%
Silty clay	2.58	36.02	20.25

### Preparation of samples

**Figure 2:**
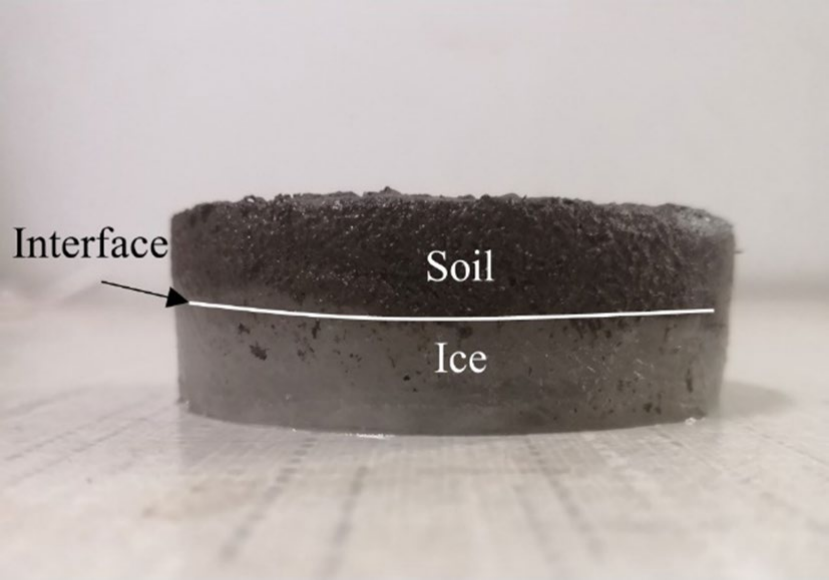
Photograph of soil-ice interface.

First, the dried soil samples were mixed with water according to different requirements, then wrapped with cling film and left for 24 h to ensure that the moisture content of the specimens remained consistent. Then, soil-ice specimens with a diameter of 61.8 mm and a height of 20 mm were prepared with a cutting ring. The specimens were formed as follows: first of all, a 10 mm thick pad was placed at the bottom of the cutting ring and covered with a layer of cling film. Then, the cutting ring was filled with distilled water and frozen at − 10 °C for 10 h. After the specimen was frozen, the ice that expands on the surface of the cutting ring was scraped off with a blade and the pad under the cutting ring was removed. Subsequently, the prepared filler is filled into the cutting ring in layers. After the soil sample was filled, the specimen was placed in an environmental box. The temperature was then set to − 10 °C and frozen for 5 h to allow the specimens to be formed, and the formed specimens are shown in Fig. 2.

### Test procedure and scheme

The molded soil-ice specimen was placed into the cooled shear box. The shear box was then wrapped with an insulating film, leaving only the top surface as a channel for heat transfer. The specimen wrapped with the insulating film was placed in the environmental box, and the temperature was controlled to reach the designed shear temperature so that the specimen starts to heat from top to bottom. Once the temperature at the soil-ice interface of the specimen reached the design value, the shear test can be started. To ensure that the test temperature at the soil-ice interface reaches the shear design value, a comparison specimen was present during the test. Except for the one-meter thermometer buried at the soil-ice interface, all the operations of the comparison specimen were the same as those of the test specimen. When the temperature of the comparison specimen reached the shear design value, the shear test can be started.

The initial moisture contents of the specimens were determined to be 9.0%, 16.0%, and 23.0% according to the corresponding moisture contents of the less-ice, more-ice, and ice-rich permafrost soils, respectively. To simulate the influence of the site environment on the shear strength of the soil-ice interface, the forming temperature was set to − 10 °C, and the shear temperatures were set to − 10, − 5, − 2, − 1, and 0 °C. Considering the sensitivity of the soil-ice interface to temperature, the rapid-shearing test was applied in this study. The normal stresses were determined as 100, 200, 300, and 400 kPa, and the shear speed was set to 0.8 mm/min. The test scheme is shown in Table 2.

**Table 2:** Orthogonal test scheme.

Soil	Moisture content/%	Shear temperature/°C	Normal stress/kPa
Silty clay	9, 16, 23	− 10, − 5, − 2, − 1, 0	100, 200, 300, 400

### Test Apparatus

**Figure 3:**
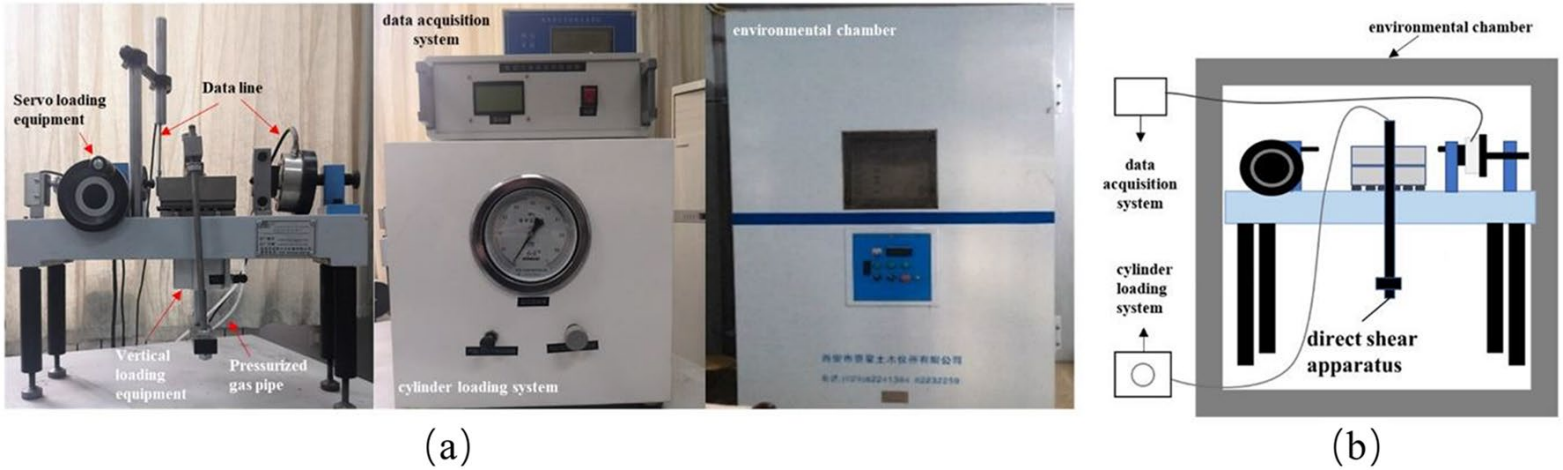
Frozen soil direct shear apparatus: (**a**) direct shear apparatus; (**b**) data acquisition system and cylinder loading system; (**c**) environmental chamber; (**d**) Diagram of test equipment.

The test apparatus consists of a direct shear apparatus, an environmental chamber (accuracy: ± 0.5 °C), an automatic data acquisition system, and a cylinder loading system, as shown in Fig. 3. The sample preparation and shear test were carried out in the environmental chamber. The stress during shearing is automatically recorded by the data acquisition system, and the positive stress is applied by the cylinder loading system.

## Results and discussion

### Shear strength of the soil-ice interface

#### Effect of shear temperature

**Figure 4:**
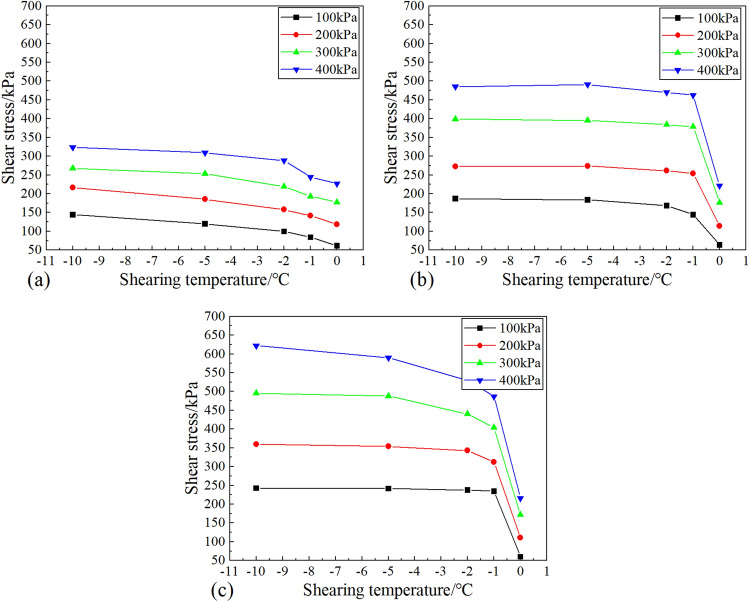
Effect of shear temperature on the shear strength of soil-ice interface: (**a**) moisture content 9.0%; (**b**) moisture content 16.0%; (**c**) moisture content 23.0%.

**Figure 5:**
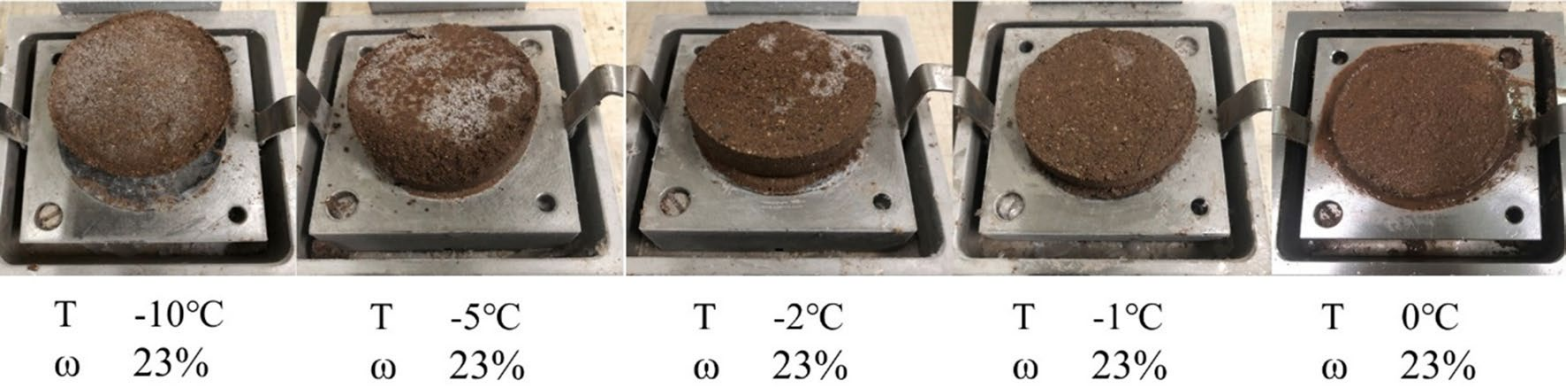
Morphology of the specimen after shear failure at different shear temperatures.

Figure 4 shows that the shear strength of the soil-ice interface decreases with the increase in temperature, which can be divided into two stages: the slowly decreasing stage and the rapidly decreasing stage. When the temperature increased from − 10 to − 2 °C, the strength of the sample decreased slowly. The strength decreases rapidly as the temperature rises from − 2 to 0 °C. This indicates that − 2 °C is the temperature threshold for the strength of the soil-ice interface. This phenomenon becomes more pronounced with increasing moisture content. Figure 5 presents the morphology of shear failure of the specimen at different temperatures when the initial moisture content is 23.0%. In the figure, T represents the temperature; ꞷ represents the moisture content. It can be found that the ice crystals on the surface of the specimen gradually decrease as the temperature rises. When the temperature was − 10℃, the whole surface of the specimen was covered with ice crystals, and when the temperature increased to − 2℃, the number of ice crystals decreased significantly. And when the temperature reaches 0 °C, the specimen starts to thaw and there is free water seepage around the specimen. In addition, it can be found that the shear failure surface is the soil-ice interface when the temperature is − 10 °C, and the failure of the soil-ice layers presents brittle damage.

The phenomenon observed from the shear test illustrated ice content plays a crucial role in the whole process of strength change with the increase in temperature.

#### Effect of initial moisture content

**Figure 6:**
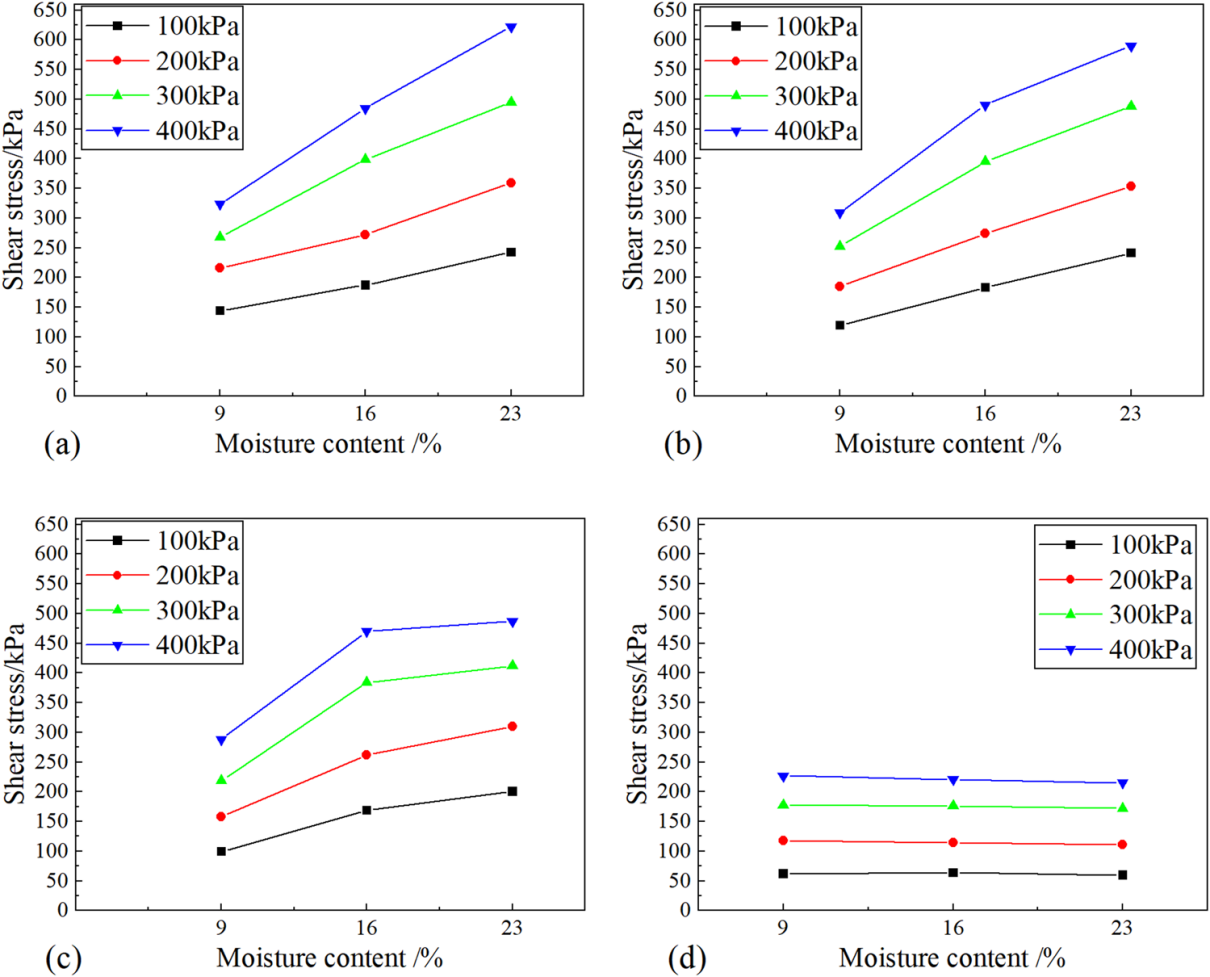
Effect of moisture content on shear strength of soil-ice interface at different shear temperatures:(**a**) − 10 ℃; (**b**) − 5 ℃; (**c**) − 2 ℃; (**d**) 0 ℃

From Fig. 6, it can be seen the shear strength of the soil-ice interface at different temperatures varies with the increase of moisture content, and the strength at 0 °C is much smaller than the strength value at negative temperature. When the shear temperature is negative, the soil-ice interface strength increases with the increase of moisture content.

**Figure 7:**
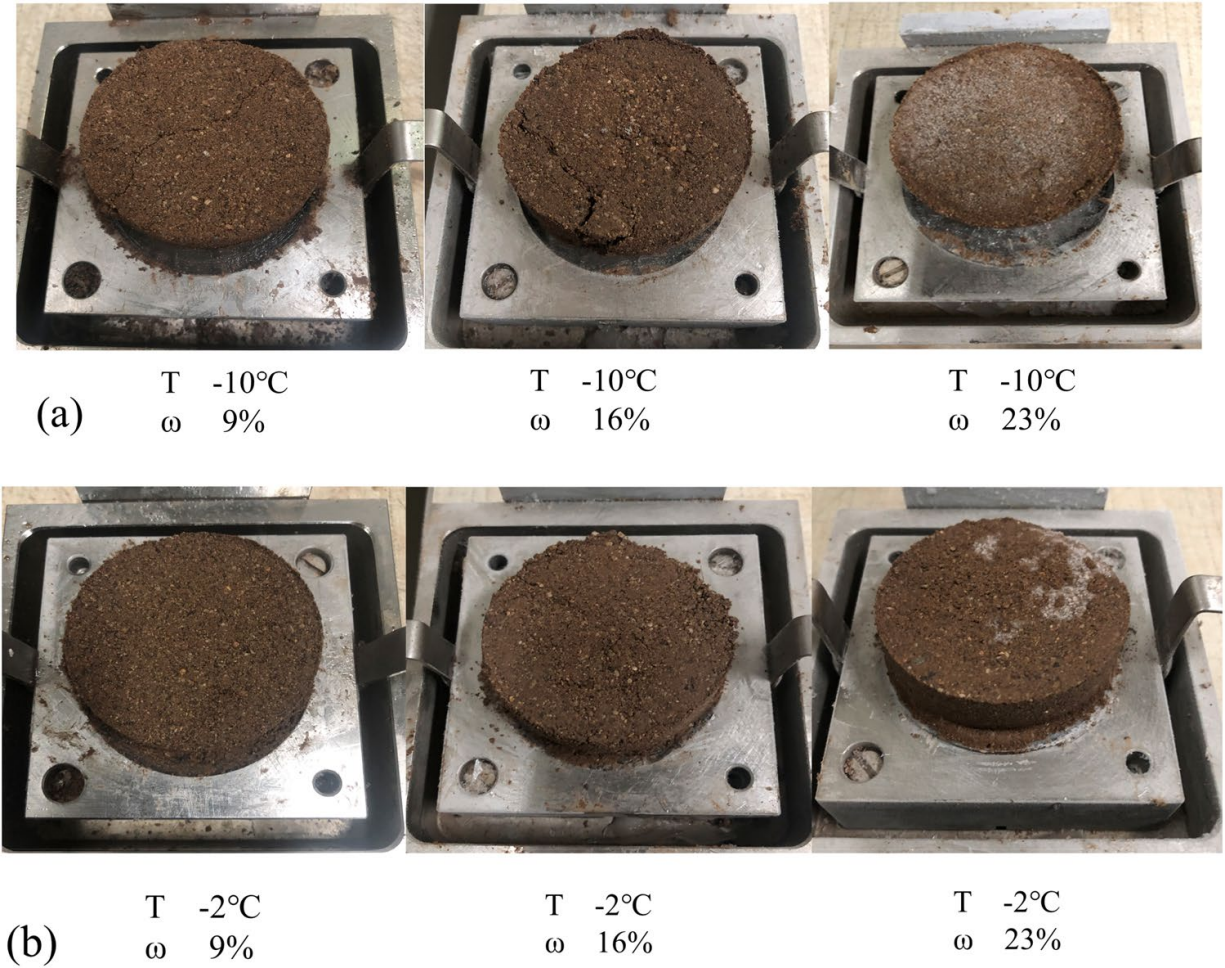
Morphology of specimens after shear failure at different moisture contents: (**a**) shear temperature of − 10 °C; (**b**) shear temperature of − 2 °C.

Figure 7 presents the morphology of shear failure of the specimen at different moisture content for the shear temperatures of − 10 °C and − 2 °C. From the chart, it can be seen that the ice crystals on the surface of the specimen increase with the growth of moisture content, which indicates that the change of strength may be related to the ice content. At lower temperatures (≤ − 5 °C), the shear strength increases nearly linearly with the growth of moisture content, and the trend is more pronounced at a temperature of 10 °C than at − 5 °C. At higher temperatures (> − 5 °C), the strength of the specimens first increased rapidly and then increased slowly. Comparing the shear failure states of the specimens in Fig. 7 at − 10 °C and − 2 °C, it can be found that the number of ice crystals on the surface of the specimen at − 2 °C is significantly less than that at − 10 °C. This indicates that the strength of the soil-ice interface is influenced not only by the initial moisture content but also by the temperature. The shear strength of the soil-ice interface decreases with increasing moisture content at a shear temperature of 0 °C. The strength of the soil-ice specimens varied from 1.73 to 14.24 kPa at different moisture contents. At 0 °C, the strength of the specimen is mainly determined by the upper soil, which is the reason why the change of strength is small. At this time, the water in the soil mainly exists in the form of free water, making the strength of the specimen change similar to that of thawed soil.

### Relationship between strength and unfrozen water content

From the test results, the effects of both shear temperature and moisture content on shear strength are related to the state and distribution of water. The strength of the specimen at 0 °C decreases with increasing moisture content, which is similar to that of the thawed soil. Changes in the unfrozen moisture content and ice content of the soil under negative temperature are important factors affecting various other changes. In turn, the change of strength is a comprehensive reflection of various physical changes. At present, the unfrozen moisture content is a physical quantity that can be quantified more accurately. Therefore, this paper reveals the variation rule of soil-ice interface strength based on the unfrozen moisture content prediction model.

As we all know, unfrozen moisture content is closely related to temperature and the initial moisture content. Based on a lot of experiments, a series of prediction models of unfrozen moisture content with temperature have been proposed by some researchers^21^. In this paper, the curves of unfrozen moisture content with temperature for powdered clay at the initial moisture content of 23.0%, 16.0%, and 9.0% were plotted using the Anderson prediction model^22^, as shown in Fig. 10. And the formula of the power function model is as follows.

1 $$W_{{\text{u}}} = \left\{ {\begin{array}{*{20}c} {a( - T)^{ - B} \, T < T_{{\text{f}}} } \\ {W_{0} \, T \ge T_{{\text{f}}} } \\ \end{array} } \right.$$

where $W_{{\text{u}}}$ Represents unfrozen moisture content; $T$ is temperature; $W_{0}$ is the initial moisture content, and $b$ are soil parameters; $T_{{\text{f}}}$ is freezing temperature.

**Figure 8:**
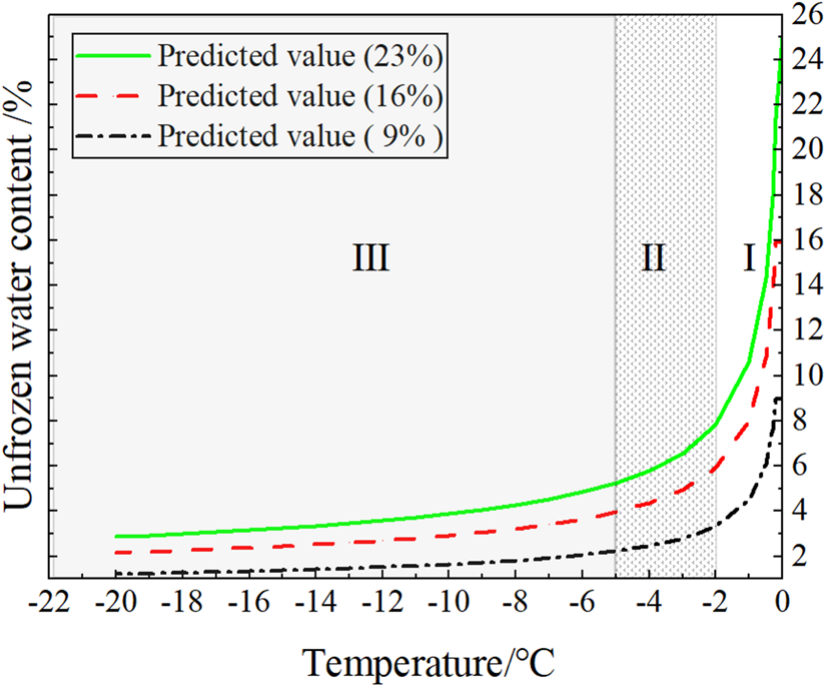
Changes in unfrozen moisture content in silty clay.

From Fig. 8 it can be seen that the unfrozen moisture content with temperature can be divided into three phases: the Severe phase transition zone (I), the transitional phase transition zone (II), and the frost zone (III)^23^. It can be noted that the change in unfrozen moisture content is most pronounced in stage I and stage II. And the shear strength of the specimens in this paper also changed very significantly in these two stages, which indicates that the unfrozen moisture content is closely related to the strength change of the specimens. In addition, it can also be found in Fig. 8 that the unfrozen moisture content increases with the increase of initial moisture content. This explains the reason why the strength increase range decreases with the increase of moisture content of the specimen when the temperature is − 2 °C. At negative temperatures, the ice content increases with the initial moisture content, and the increase in ice content significantly increases the strength of the specimens. However, the increase in initial moisture content will also significantly improve the content of unfrozen water in the specimen, which will reduce the strength of the specimen. Therefore, when the moisture content exceeds 16% at a temperature of − 2 °C, the increased range of strength decreases significantly. To further investigate the mechanism of strength change at the soil-ice interface, the variation of the internal friction angle and cohesion of the specimen at different temperatures and water contents are analyzed below.

### Shear index of the soil-ice interface

#### Effect of Shear Temperature

##### Cohesion

**Figure 9:**
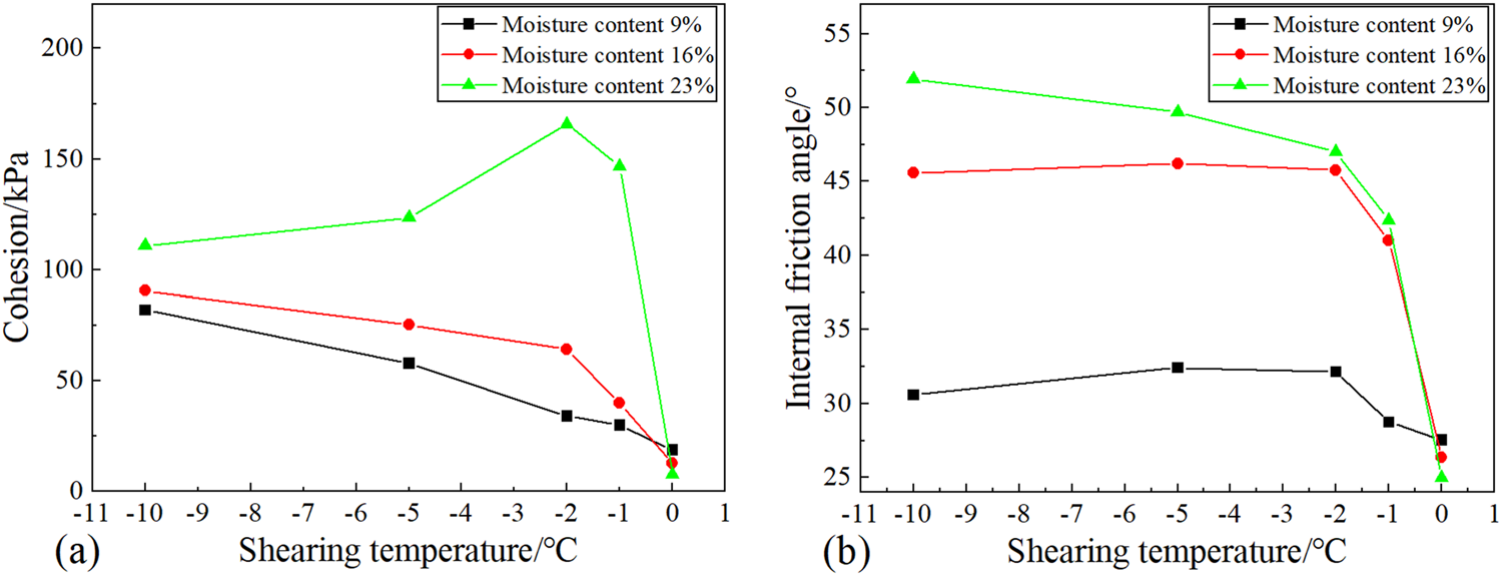
Effect of shear temperature on the shear index of soil-ice interface: (**a**) Cohesion; (**b**) Internal friction angle.

From Fig. 9a, it can be seen that the cohesion of the samples behaves differently in different moisture contents. When the moisture content was 9.0% or 16.0%, the cohesion increases rapidly with the decrease of temperature and then increases slowly. At a moisture content of 23.0%, the cohesion decreased by 54.59 kPa instead of increasing as the temperature decreased from − 2 to − 10 °C. In addition, it can be noted that the cohesion increases rapidly with the decrease of temperature from 0 to − 2 °C under different moisture contents. When the moisture content was 23.0%, the cohesion increased from 7.68 to 165.84 kPa as the temperature decreased from 0 to − 2 °C, which increased by 95.37%. From Fig. 9a, it can be found that the change in cohesion is most significant in stage 2, while it is less variable in stage 4 and stage 3. This indicates that the variation of cohesion corresponds to the variation of unfrozen water content. When the moisture content is 9.0% and 16.0%, the unfrozen moisture content decreases as the temperature drops, which makes the ice cementation and capillary cohesion of the specimen increase, so the cohesion of the specimen increases. Moreover, the unfrozen moisture content changes most significantly in stage 2, so the cohesion has a significant change at this stage. When the moisture content was 23.0%, the decrease of cohesion at lower temperatures (< − 2 °C) may be related to the change in soil structure ^24,25^. At a moisture content of 23.0%, as the temperature drops from 0 to − 2 °C, a large amount of unfrozen water freezes into ice causing the pores between the soil particles to be filled. Subsequently, as the temperature continues to drop, the pore ice content increases further. The volume expansion caused by the unfrozen water freezing into ice at this time will squeeze the soil particles and force them to move. And the mutual slip between soil particles disrupts the cementation between ice and soil particles, which weakens the contribution of ice cementation to cohesion, and thus cohesion decreases with temperature. It should be noted that due to the low content of unfrozen water when the temperature is lower − 2 °C, the cohesion is mainly influenced by the ice cementation effect, and the role of matrix suction can be ignored. It can be found that the variation of cohesion with temperature is closely related to the state of water in the specimen.

##### Internal friction angle

From Fig. 9b, it can be seen that the internal friction angle decreases slowly at first and then decreases rapidly with the increase of shear temperature. The internal friction angle remained unchanged as the shear temperature increased from − 10 to − 2 °C when the moisture content did not exceed 16.0%, while it decreases by 4.91° for the moisture content of 23%. As the shear temperature rises from − 2 to 0 °C, the internal friction angle of the specimens decreased by 4.62°, 19.43°, and 22.00° when the moisture content was 9%, 16%, and 23.0%, respectively. It can be found that the variation of the internal friction angle is also closely related to the variation of unfrozen water content.

When the moisture content is 9% or 16%, the generated pore ice does not fill the pores, while the pressure on the soil particles grows as the pore ice increases. The higher the pressure between soil particles, the higher the coefficient of friction, so the internal friction angle varies with the ice content. At a moisture content of 23.0%, the ice almost fills the pores of the soil. The increase of ice content at this time may make the pressure in the soil exceed the limit value, which causes displacement of the soil particles as well as ice particles. And the slippage between the particles will significantly increase the mechanical bite force between the grains. Therefore, when the moisture content is 23.0%, the lower the temperature, the greater the internal friction angle.

#### Effect of initial moisture content

##### Cohesion

**Figure 10:**
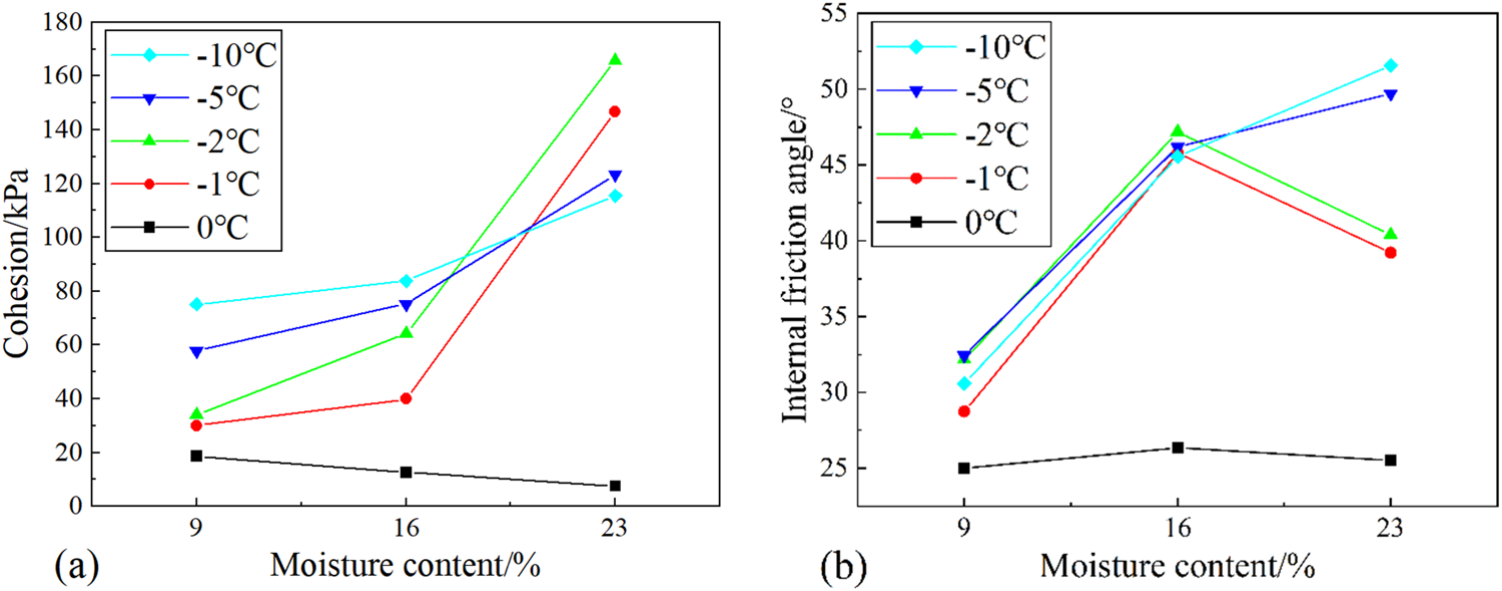
Effect of moisture content on the shear index of soil-ice interface: (**a**) cohesion; (**b**) internal friction angle.

From Fig. 10a, it can be seen that the cohesion has different trends at different temperature conditions as the moisture content increases. When the shear temperature was 0 °C, the cohesion decreased from 18.81 to 11.59 kPa with an almost linear development as the moisture content increased from 9.0 to 23.0%. And when the shear temperature is negative, the cohesion tends to increase with the increase of moisture content. When the shear temperature is − 1 °C, the cohesion increased from 30 to 39.9 kPa with the increase in moisture content from 9 to 16%, which is an increase of about 33%. And the cohesion increased from 39.9 to 146.79 kPa with the increase of moisture content from 16.0 to 23.0%, which is an increase of about 267.4%. At the shear temperature of − 2 °C, the cohesion increased from 34 to 65 kPa with the increase of moisture content from 9 to 16%, which is an increase of 88.7%. And the cohesion increased from 65 to 165.84 kPa with the increase of moisture content from 16.0 to 23.0%, which is an increase of 158.4%. While at lower temperatures (< − 2 °C), although the cohesive strength of the specimens increased, the increased range was smaller than that at a temperature of − 2 °C and − 1 °C. It can be found that there is a threshold moisture content (i.e.16.0%) when the temperature is negative, and the cohesion of the specimen increases rapidly when the value is exceeded. In addition, the variation of cohesion with moisture content is also affected by the shear temperature, and it can be found that the higher the temperature, the greater the increase of cohesion with moisture content. A possible explanation for this might be that the growth of ice content affects the development of strength. At a temperature of − 5 °C, the cohesion only increased by about 64.1% as the moisture content rise from 16.0 to 23.0%. This is because the ice content increases as the temperature decreases, and when the ice content reaches the initial frost heave ice content of the specimen, the volume increment generated by the water turning into ice will make the soil particles slip. The displacement between soil particles will damage the ice cementation leading to the reduction of cohesion. This explains why the cohesion at − 5 °C is less than that at − 2 °C when the moisture content reaches 23.0%.

##### Internal friction angle

From Fig. 10b, it can be seen that the variation trend of the friction angle at the soil-ice interface is not the same with increasing moisture content at different temperatures. The friction angle of the soil-ice specimen hardly varies with the moisture content at a shear temperature of 0 °C. While at a negative temperature, the internal friction angle of the specimen generally tends to increase with the increase of moisture content. This is because the water in the upper soil mostly exists in the form of free water at 0 °C. Although the increase of moisture content makes the lubricating effect increase, the friction angle does not change significantly. And at a negative temperature, with the increase of moisture content, the pore ice content in the soil increases, which makes the stress between soil particles grow, so the friction angle increases with the moisture content. While the temperature is − 2℃ and − 1℃, the friction angle first increases and then decreases. This change is closely related to the unfrozen moisture content in the soil. At a temperature of − 2 °C, most of the free water in the soil is frozen into ice. At this time, the volume expansion of water into ice will make the stress between soil particles increase, which makes the internal friction angle enhanced. However, as the initial moisture content increases, although the ice content also increases, the unfrozen moisture content increases significantly at this time, which makes the lubrication between the particles greatly enhanced, so the internal friction angle of the specimen starts to decrease after the moisture content exceeds 16.0%. And the internal friction angle grows with the increase of moisture content when the temperature is − 5 °C and − 10 °C. However, when the moisture content is 23.0%, the internal friction angle increases rather than decreases with the increase of moisture content. The reason for this phenomenon is related to the occurrence of frost heave. When the moisture content reaches the starting frost heave moisture content, the soil particles slide because of the frost heave, which makes the mechanical bite force between the particles increase. Therefore, the internal friction angle increases.

### Analysis of results

#### The change mechanism of shear strength

According to the variation of shear strength and shear index under different conditions, it can be discovered that the effects of cohesion and internal friction angle on shear strength are not the same under different factors. When the moisture content is low (≤ 16.0%), the cohesion of the specimen starts to decrease with the increase in temperature, while the internal friction angle remains unchanged, and the change in the strength of the specimen is mainly due to the decrease of the cohesive force. When the moisture content is 23.0%, with the increase of temperature, the cohesion of the specimen increases and then decreases, while the friction angle decreases slowly and then decreases rapidly. At lower temperatures (< − 2 °C), the cohesion and the friction angle of the specimens increased with the moisture content, and the strength of the specimens was influenced by both together. At higher temperatures (− 2 °C ~ 0℃), the cohesion of the specimens increased slowly and then rapidly with the increase of moisture content, while the internal friction angle increased rapidly and then decreased. Although the change of cohesion was also significant, the strength of the specimens was more influenced by the internal friction angle.

### Shear failure surface of the soil-ice specimen

**Figure 11:**
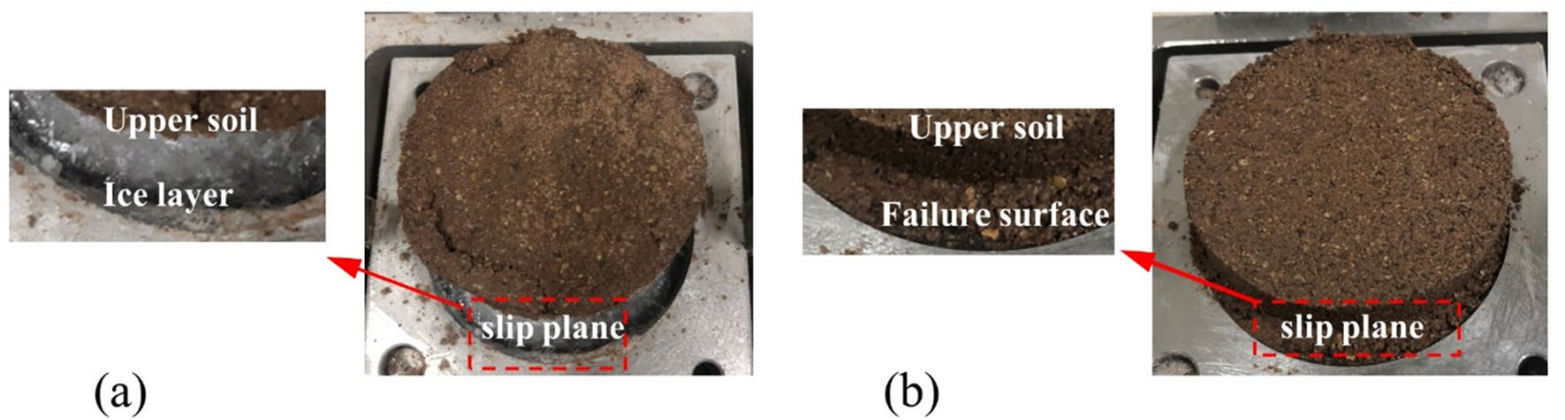
Location of shear failure surface: (**a**) soil-ice interface; (**b**) in the upper soil layer.

The results of the shear test illustrate the shear failure surface appears at the soil-ice interface and in the upper soil layer, as shown in Fig. 11. The reason for this phenomenon may be greatly related to the structure of the specimen. The direct shear test makes the location of shear failure relatively fixed, but the shear failure surface does not necessarily appear at the soil-ice interface.

The specimen consists of two parts: the ice layer and the soil layer. The strength of the specimen is determined by the strength of the ice layer, the strength of the frozen soil, and the strength of the soil-ice interfaces. In general, the strength of ice far exceeds the strength of soil, so the shear strength of the specimen is determined by the strength of the upper frozen soil layer and the soil-ice interface.

It should be noted that the test results may be influenced by the size of the specimen and the location of the soil-ice interface during shear. However, the authors still found some regularity based on the multiple sets of experiments. According to the shear test results in Figs. 5 and 7, it can be found that the failure surface appears at the soil-ice interface for a shear temperature of − 10 °C. While the shear failure surface is located above the soil-ice interface when the temperature is higher than − 5 °C. It is also found that the higher the moisture content at lower temperatures, the better the integrity of the upper soil layer is, and the upper part is a whole after shear failure. This indicates that the strength of the upper soil layer of the specimen is higher than that of the soil-ice interface when the failure surface is at the soil-ice interface. In contrast, the strength of the soil-ice interface is higher than the strength of the upper soil layer when the temperature is higher than − 5 °C.

## Discussion

The objective of this study is to evaluate the interface shear behavior and strength of the soil-ice interface under different temperatures and moisture content. The shear failure surface of samples is also evaluated based on the shear morphology of specimens. The pattern of change in the strength of the specimens at moisture content and temperature is similar to that of the general permafrost soil. The development of strength is closely related to the variation of unfrozen water content. The cohesion decreases first and then increases with increasing temperature when the moisture content is 23%. This finding is consistent with that of Du et al.^26^ who believe that the variation trend is related to unfrozen water content. According to the shear failure morphology, we found that the shear failure surface is not always in the soli-ice interface. As the temperature increases, the weak surface of the specimen moves from the soil-ice interface to the upper soil layer. This finding is consistent with that of Gao et al.^14^ who also found that the weak surface of the specimen is located in the freeze–thaw interface or upper soil layer. In addition, it can be inferred from the shear damage morphology that the strength of the soil-ice interface is greater than that of the soil layer when the temperature is above − 5 °C, while the strength of the soil-ice interface is less than that of the upper soil layer when the shear temperature is − 10 °C. This finding is consistent with that of Chen et al.^27^ who believe that the variation trend is related to unfrozen water content.

During the spring thaw season, the frozen soil is influenced by the external air temperature, and the heat energy is transferred from the surface to the interior, and the temperature near the outside is significantly higher than the temperature inside the permafrost. Therefore, the strength of the upper soil layer has been significantly decreased before the heat energy is transferred to the ice layer. At this time, the weak surface of the permafrost does not occur at the soil-ice interface but is located in the upper soil layer. From the available results and findings, it can be seen that the strength of the soil-ice interface is higher than the general permafrost at temperatures above − 5 °C. Therefore, the weak surface of the slope will not appear at the soil-ice interface during the spring thaw but will be located in the upper soil layer.

## Conclusion

According to the experimental results presented here, the following conclusions can be drawn:

1. The strength of the sample decreases with increasing temperature, and the change in strength is most significant from − 2 to − 0 °C. The strength reduction in this range is from 21.8 to 74.8%, and the higher the moisture content the more obvious this phenomenon is. The strength of the sample increases with increasing moisture content at negative temperatures, and the increase is more significant at lower temperatures. With the increase of moisture content from 9.0 to 23.0%, the strength increases by 55.1%, 48.4% and 46.8% at − 10 °C, − 5 °C and − 2 °C, respectively. The shear strength of the specimens decreased with the increase of moisture content at 0 °C due to the presence of large amount of free water.
2. The changes of cohesion and internal friction angle are closely related to the unfrozen water content, and the changes of strength are influenced by both of them together. The shear index tends to decrease with the increase of unfrozen water content, and the greater the increase of unfrozen water, the faster the decrease of both, especially in stage 2. It should be noted that when the moisture content is 23%, the cohesion and the internal friction show the alternating growth and decline trends, and the angle of internal friction has a higher influence on the strength than the cohesion.
3. When the temperature is higher than − 5 °C, the specimen failure surface is located in the upper soil layer, slightly above the soil-ice interface. At this time, the strength of the soil-ice specimen is determined by the strength of the upper soil layer, and the strength of the soil-ice interface is greater than the strength of the soil layer. When the shear temperature is − 10 °C, the soil-ice interface is the shear failure surface. At this time, the strength of the soil-ice interface is smaller than the strength of the upper soil layer.

## Data Availability

Some or all data, models, or codes that support the findings of this study are available from the corresponding author upon reasonable request (list items).
